# Reducing HIV/STI Risk Among Adolescents Aged 12 to 14 Years: a Randomized Controlled Trial of Project Prepared

**DOI:** 10.1007/s11121-021-01203-0

**Published:** 2021-02-19

**Authors:** Laurie J. Bauman, Dana Watnick, Ellen Johnson Silver, Angelic Rivera, Jamie Heather Sclafane, Caryn R. R. Rodgers, Cheng-Shiun Leu

**Affiliations:** 1 Preventive Intervention Research Center, Department of Pediatrics, Albert Einstein College of Medicine, 1300 Morris Park Avenue VE6B25, Bronx, NY 10461 USA; 2 Department of Pediatrics, Albert Einstein College of Medicine, Bronx, NY USA; 3 Division of Public Health, William Paterson University, Wayne, NJ USA; 4 Mailman School of Public Health, Columbia University, New York City, NY USA

**Keywords:** Adolescents, HIV prevention, STI prevention, Randomized controlled trial

## Abstract

Despite calls for evidence-based HIV/STI prevention programs for youth aged 12 to 14 transitioning to adolescence, few effective programs exist. In a two-group intent-to-treat randomized trial in the Bronx, NY, 397 participants were randomly assigned to Project Prepared or an attention control, TEEN. Participants completed surveys at baseline, 6 months, and 12 months. Prepared had two components, an 11-session program and a 3-week internship. Content covered sexual risk behavior, social cognitions, gender norms, relationships, and resilience. TEEN built communication skills and had the same intensity and structure as Prepared but no sexual content. In both, boys and girls were trained together in mixed groups of ~ 11 teens. Primary outcomes were HIV knowledge, self-efficacy, condom outcome expectancy, and behavioral intentions. Secondary outcomes were relationship expectations and endorsement of risky gender norms. Generalized estimating equation analyses showed youth randomized to Prepared had significant improvements compared to TEEN at T2 in HIV knowledge, sexual self-efficacy, and outcome expectancy for condom use. At T3, there were significant differences favoring Prepared in outcome expectancy for condom use, sexual self-efficacy, and intention for partner communication about HIV/AIDS or STIs. Analyses by gender showed program effects in both boys (intention to talk to a partner about condom use, abstinence self-efficacy, sexual self-efficacy, and condom outcome expectancy) and girls (gender norms, and abstinence outcome expectancy). Prepared effectively reduced risk in young adolescents. ClinicalTrials.gov ID: NCT01880450, Protocol ID: 2008-551

**Supplementary Information:**

The online version contains supplementary material available at 10.1007/s11121-021-01203-0.

## Background

### Problem of Sexual Risk in Early Adolescents

Over half (57%) of US high school students engage in sexual intercourse by the time they graduate (Witwer et al., 2018). Early sexual initiation increases risk for HIV/STIs (CDC 2015) and unintended pregnancy (Heywood et al., 2015). Few (6%) high school students report sexual debut before age 13; males more than females report early coitus (8.3% vs. 3.1%) and Black males report the highest rate of early coitus (24%) compared to Latino (9.2%) and white (4.4%) males (Kann et al., 2014).

### Predictors of Sexual Risk in Adolescents

Many factors predict sexual risk in adolescence (Brown and Larson, 2009; Kirby et al., 2005; Kourtis et al., 2006; Parkes et al., 2011; Widman et al., 2016). Kirby and Lepore 2007’s review of over 400 studies identified several hundred potentially important variables. Older age, male gender, economic disadvantage, Black race, and Hispanic ethnicity predict sexual activity (CDC, 2015). Family characteristics can be both protective (close parent relationship; good parent-teen sexual communication (Kirby and Lepore, 2007; Parkes et al., 2011; Widman et al., 2016)) and risk factors (single-parent family structure; low parental education (Silver and Bauman, 2006)). Sexual risk is higher in youth who use substances, have poor academic performance, and poor psychosocial adjustment, and lower in those with positive school attitudes, college aspirations, and participate in after-school activities (Kirby and Lepore, 2007; Silver and Bauman, 2006). Developmentally, peer relations become more salient in adolescence (Brown and Larson, 2009). Social acceptance and social status become more important and peer pressure and willingness to conform are especially strong (Brown and Larson, 2009). Thus, attitudes and values of romantic partners, friends, and other peers and beliefs about sexual activity of close friends are influential (Kirby and Lepore, 2007).

Emotional competence, resilience, and self-efficacy beliefs are also associated with risk, as are structural factors described in theories of gender and power (DePadilla et al., 2011). Gender norms, the attitudes and behaviors that a culture or society associates with each sex, are important determinants of health behavior (Fleming and Agnew-Brune, 2015). Gender norms about sexual behavior for males typically suggest that they should be knowledgeable and in control and should initiate and pursue all sexual opportunities, while female sexuality is relationship-centered and associated with emotional commitment; women are expected to resist sex in situations where such motives are not sufficiently powerful (Marston and King, 2006; Silver and Bauman, 2014; Tolman et al., 2003). Risky gender norms may conflict with safer sex practices; however, there is little systematic research on this topic among young adolescents (Silver and Bauman, 2014).

Relationship beliefs and expectations also impact sexual risk (Ellen et al., 2002; Rosengard et al., 2005; Misovich et al., 1997; Rosenthal et al., 1998). Being in a committed and loving relationship can increase risk for unprotected sex because condom use is lower in committed than casual relationships (Ellen et al., 2002; Rosengard et al., 2005, Bauman and Berman, 2005). Condom use is inconsistent with ideals of romance, trust, and love, especially for girls (Rosenthal et al., 1998; Bauman and Berman, 2005). Although young adolescents may not be in this kind of relationship yet, many are involved in the precursors, including being in love (Montgomery and Sorell, 1998; Bauman and Berman, 2002). Serious adolescent relationships are fairly short-lived, many engage in “serial monogamy (Misovich et al., 1997), and many youth have concurrent partners during an ostensibly exclusive relationships (Rosenberg et al., 1999); thus, adolescents may expose themselves to considerable risk.

### Theoretical Frameworks

Research on HIV prevention among adolescents has employed several different theoretical frameworks. Social Cognitive Theory emphasizes efficacy expectations and outcome expectancies (Bandura, 1986; Bandura, 1989). The Theory of Reasoned Action posits that intention to perform a behavior is key to whether it will occur, which is related to perceptions of how the action might be perceived by others (“subjective norms”) (Ajzen and Fishbein, 1980; Bandura, 1989). The Theory of Planned Behavior extends the Theory of Reasoned Action by adding degree of perceived personal control over it (Ajzen, 1991). Sexual risk reduction programs based only on these cognitive theories have small, short-term effects, prompting researchers to apply socio-ecological perspectives (Bronfenbrenner, 1977; Miller et al., 2000; Kotchick et al., 2001; Coates et al., 2008; Protogerou and Johnson, 2014; Atkiss et al 2011; Mason-Jones et al., 2016) that emphasize positive youth development, gender norms, relationship status, and developmental stage. Eco-developmental Theory (Perrino et al., 2000; Pantin et al., 2004) adds a developmental perspective—the person and context change over time (Pantin et al., 2004). Adolescents experience many cognitive changes in the ways they view themselves and the world, some of which affect their processing of health-related messages and their engaging in risk behaviors for HIV/STIs (Serovich and Greene, 1996). A cognitive phenomenon called the “personal fable” has been proposed as a partial explanation for the tendency of adolescents to engage in risky behavior (Serovich and Greene 1996). Adolescents display egocentric thinking and believe both in the uniqueness of their emotional experience and in their immortality or invincibility. Elkind (1967) suggests that egocentricism results from the transition into formal operational stages of thought, whereas Vartanian (2000) believes that the personal fable reflects changes in social perspective-taking and interpersonal understanding.

Positive youth development (PYD) is an effective strategy to promote adolescent sexual health. Developmental assets such as family connectedness and communication, parental monitoring, school connectedness, cognitive and social competence, belief in the future, and self-efficacy are associated with sexual health (Catalano et al., 2010; Gloppen et al., 2010; House et al., 2010a; House et al., 2010b). Accurate, evidence-based sex education combined with PYD approaches can build resilience (Gavin et al., 2010). Advocates of the assets-based approach have noted the critical importance of social/relational factors for sexual behavior and recommend that these should be addressed in preventive interventions (Romeo and Kelley, 2009).

From the literature on adolescent sexual risk behavior and the theories that guide interventions, we identified the content of Project Prepared. Because young people 12–14 years of age have not yet initiated sexual risk behavior, we chose a prevention approach rather than a behavior change approach. Thus, the intervention provided the tools and resources young people would need to guide choices in their future sexual behavior. These were (1) knowledge about sexuality, how STIs are transmitted, and pregnancy prevention; (2) strategies to reduce risk (e.g., condom use and contraception, refusal skills, condom use negotiation); (3) cognitions (e.g., self-efficacy) to assure confidence in using risk reduction strategies; (4) understanding of gender norms and how they influence sexual behavior; (5) understanding of how relationship factors and power differentials influence behavior and skills in partner communication; (6) motivation to adopt risk reduction behaviors; and (7) intention to engage in safer sex behaviors.

### Experience of Interventions with Youth Aged 12–14

Interventions for 12–14 year olds demonstrate mixed results (Coyle et al., 2004; Mason-Jones et al., 2016; Nelson et al., 2016). “For Keeps” (Borawski et al., 2005) increased HIV-related knowledge and decreased intentions to have intercourse but did not influence sexual initiation or condom use. “Postponing Sexual Involvement” (Kirby et al., 1997) demonstrated a few small effects at 3 months; none was sustained over time, and the program did not delay age at first coitus. Others have found positive effects on HIV knowledge but not on self-efficacy, intention, attitudes, delayed sexual initiation, or condom use (Clark et al., 2005; Flay et al., 2004). “Wise Guys” (Gruchow and Brown, 2011) showed positive effects on knowledge, attitudes, and behavior but was designed for boys only; others have shown effects on knowledge and efficacy only for girls (Noia and Schinke, 2007). “Focus-on-Kids” (Gaydos et al., 2008) showed early effects on condom use and condom use intentions in 9–15-year-old youth that did not persist. Jemmott, Jemmott & Fong (1998) showed decreased intercourse and increased condom use in 6th and 7th graders, but only for sexually experienced teens.

**Fig. 1:**
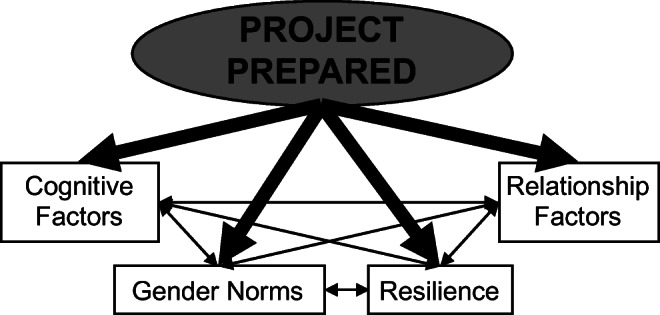
Project Prepared conceptual model

Project Prepared is an intensive and comprehensive group-based intervention for adolescents aged 12–14. Our conceptual model is based on the literature and theory (Fig. 1). Each conceptual element determined the content of the intervention curriculum. **Cognitions** were drawn from Social Cognitive Theory and included HIV knowledge, abstinence self-efficacy, and abstinence and condom use outcome expectancies. For **gender norms**, a construct in developmental and social-ecological theory, we helped youth recognize the generally held beliefs about what behaviors are appropriate based on gender, and how they influence having sexual intercourse, having sex by a certain age, or having multiple partners. Resilience is personal attributes that are critical for overcoming life obstacles. We focused general self-efficacy, relatedness (including trust and support), and emotional reactivity and relied on Positive Youth Development for curriculum exercises. **Relationship factors** are beliefs about romantic relationships that can increase the likelihood of early sexual initiation or unprotected sex, and how feelings of love and trust and assumptions of monogamy can lead to risky behavior. The entire curriculum was informed by developmental theory and how 12–14-year-olds learn and behave (e.g., egocentric thinking, short attention span, limited use of role plays). The conceptual model guided the main hypotheses, that those randomized to Prepared, compared to the control group, would have improved knowledge and social cognitions; increased recognition of risky gender norms; more resilient attributes; healthier relationship beliefs; and stronger intentions to engage in safer sex behavior (abstinence, talking to partners about HIV/AIDS, condom use).

## Methods

**Fig. 2:**
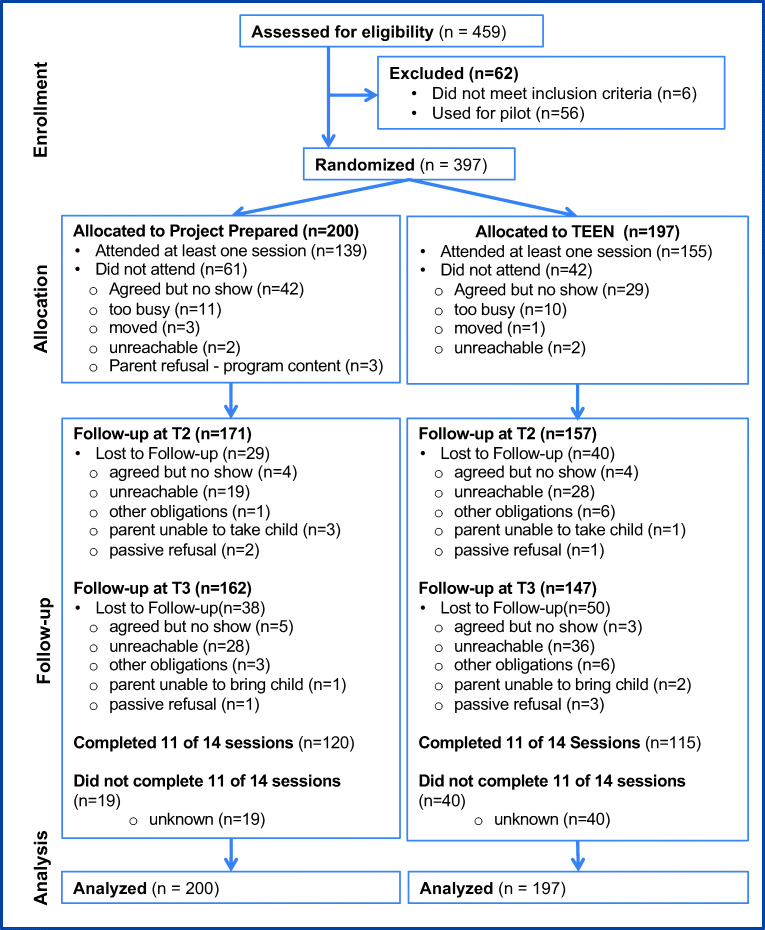
CONSORT diagram of participant flow

A two-group intent-to-treat randomized controlled trial of Project Prepared was implemented in the Bronx, NY. Adolescents were recruited from primary care practices at Montefiore Medical Center (MMC) over 3 years beginning in 2011 (see Fig. 2). These primary care practices provide routine preventive care as well as manage youth with chronic health conditions. Each year, 2 cohorts of 66 youth were recruited and randomized to Prepared (the experimental condition) or TEEN (the attention control condition) (Bauman et al., 1997). Primary outcomes were HIV knowledge, self-efficacy, condom outcome expectancy, and behavioral intentions. Secondary outcomes were relationship expectations, resilience, and identifying gender norms.

### Eligibility, Recruitment, and Randomization

Participants were identified using MMC’s computerized patient database. Patient names, addresses, and age were generated with no health data to preserve confidentiality. Parents of youth received a letter from MMC indicating their child would be invited to complete a computer-assisted questionnaire called the “Teen Lifestyle Survey” (TLS) at The Preventive Intervention Research Center at the Albert Einstein College of Medicine, unless parents opted out. Of 981 initial invitations, 247 were undeliverable and 38 parents opted out. The remaining 696 were mailed an invitation to TLS; another 26% were undeliverable and 54 parents refused (11%). Parent consent and youth assent were obtained for participation in TLS. The target sample size of 400 was selected to detect small to moderate effects assuming a 20% loss to follow-up. Eligibility criteria were (1) age 12–14 inclusive; (2) physical ability to travel independently to the program site; (3) 5th grade reading ability on the reading subtest of the Wide-range Achievement Test (Jastak and Wilkinson, 1984) (required to read program materials and complete TLS); and (4) spoke and understood English. A total of 397 youth was enrolled and randomized after completing TLS and having eligibility verified. Youth were enrolled over the course of 3 years in 6 cohorts averaging 66 adolescents each to assure ~ 30 teens per condition in each cohort. Following randomization, youth were re-enrolled with a new parental consent and child assent describing the program being offered and requirements for participation. To assure between-group equivalence in age and gender, each cohort of ~ 66 was stratified at recruitment by gender and age into 6 groups of *n* ~ 11 youth: females ages 12, 13, and 14; males ages 12, 13, and 14. Before recruiting each cohort, assignment lists were created; the field supervisor assigned each eligible adolescent to the next slot in their subgroup.

### Intervention

**Project Prepared** had two parts. An 11-week interactive classroom-style intervention was followed immediately by a 3-week internship, for a total of 14 sessions. The internship was a pedagogical tool in which participants used what they had learned in Prepared and taught it to others. The ~ 33 youth randomized to both TEEN and Prepared were divided in half for an ideally sized group of 12–15 participants. For the internship, each group of 12–15 was split again into 2, each producing their own poster presentation. The internship began the week following the classroom intervention. The goal of the internship was for participants to use their new expertise through creating a poster presentation to educate other teens about the topics in the Prepared program. In the first week, participants chose a poster topic; in the second week, they designed their poster and practiced their presentation; and in the third week, a panel of experts in sexuality education attended their poster presentations and gave feedback. Youth then presented the poster to peers who voted to select the winning poster presentation; winners each received a $10 gift card.

Prepared met once weekly for 2¼ hours. It included large and small group discussions, separate gender group discussions, icebreakers, and role plays. It covered information about sexuality, including STIs, HIV, and condom use; awareness of gender and relationship norms; perceived and actual peer pressures that influence the ways in which people behave and how these can increase sexual risk-taking; and skills in communication, decision-making, and building healthy relationships. Risk reduction strategies included abstinence; correct, consistent condom use; limiting number of partners; and recognizing healthy/unhealthy relationships. Adolescents received $15 each week for participating. Each Prepared group was led by one facilitator, with an assistant facilitator who documented fidelity to the intervention manual and assisted with logistics (e.g., attendance, food, pay) and small group work. The internship was run by 4 facilitators; each was assigned to one of the 4 poster groups.

**TEEN** was similarly structured as an 11-week communication and social skills program, and a 3-week internship (14 total sessions) in which adolescents created a poster presentation to teach peers about communication and social skills. TEEN was a perfect attention control intervention: it was successful in enhancing self-esteem and reducing distress (Bauman et al., 1997), and it was as intensive as Prepared, but had no HIV/STD, gender norm, or relationship content.

All facilitators had strong skills in sexuality education, HIV/STI risk reduction, and group facilitation with degrees in health education, public health, or health psychology, and were experienced in working with adolescents. They were trained in cultural competence, the intervention protocol, protocol manuals, and to adhere strictly to the protocols.

Acceptability of both programs was high. Satisfaction data for both programs was collected at the end of each cohorts. The 39-item survey asked about comfort with the program, development fit, satisfaction with the content, and satisfaction with the facilitator. Satisfaction ratings for both programs were very high; this ceiling effect precluded formal analysis, but visual inspection showed no gender, race, or program differences.

### Data Collection and Measures

Adolescents completed data collection three times between the years 2011 and 2015: at T1, baseline; T2, 6 months later, after the programs were completed; and T3, 1 year after baseline via self- and interviewer-administered tools. Participants completed surveys at private Computer-Assisted Personal Interview (CAPI) terminals. Trained interviewers administered a survey on school attendance, family structure, and personal background. Youth received $25 for completing each interview (Table 1).

**Table 1:** Measures

Construct/measure	Description	Notes
Cognitive factors: social cognitions		
HIV knowledge (Volpe et al. 2007)	18 items, answered yes, no, do not know	Means are reported
Abstinence self-efficacy (Diiorio et al. 2002, 2006)	12 items answered on a 7-point scale, “not sure at all” to “completely sure”	Items included staying out of situations that lead to pressure to have sex
Abstinence outcome expectancy (Diiorio et al. 2001,2002, 2006)	19 items answered on a 5-point Likert scale. Alpha reliability was .85	The perceived consequences of enacting risk reduction behavior
Sex-related self-efficacy (Diiorio et al., 2001)	12 items answered on a 7-point scale “not sure at all” to “completely sure”	Self-efficacy for safer sex behavior, e.g., using a condom, negotiating condom use
Condom use outcome expectancy (Diiorio et al., 2001)	9-item measure answered on a 5-point Likert scale; summed (higher = more favorable attitudes)	Self-evaluative, physical, and social expectancies of condom use (e.g., I will feel more responsible if I use a condom)
Cognitive factors: behavioral intentions		
Behavioral intentions (Diiorio et al., 2002)	yes/no items	e.g., intention to have sex, limit sexual partners, talk to partners about HIV, use condoms every time
Behavioral intentions (CAPS, 1994)	2 items answered on a 5-point scale from “I would definitely” to “I would definitely not”	Whether they would refuse to have sex without a condom and whether they would insist even if partner disagreed
Behavioral intentions (developed for the study)	Future plans, answered on a 5-point scale from “never” to “will definitely do frequently”	Having sexual intercourse, having condoms available, using condoms, talking to sexual partners
Gender norms		
Gender norms (Silver et al., 2014)	6 items answered on a 4-point Likert scale; parallel versions for boys and for girls; higher = higher endorsement of risk behaviors for that gender	Having sexual intercourse, having sex by certain ages, or having multiple partners make someone “cool” or “popular”
Relationship factors		
Romantic Beliefs Scale (Marin,Coyle, Gomez, Carvahal & Kirby, 2000)	5-items answered on a Likert scale	Adolescent beliefs about a romantic love relationship., e.g., If I love someone, I know I can make the relationship work, despite any obstacles
Relationship expectations (Watnick et al., 2011)	12 items answered on a 4-point Likert scale; two subscales. Relationship monitoring & control; emotional openness & availability. Items summed; higher = more positive expectations	e.g., when I have a girl/boyfriend… s/he will want to know where I am at all times;…“I will tell my partner how I feel about her/him”
Resilience		
Resiliency scales for adolescents, profile of personal strengths (Prince-Embury, 2007)	Both raw scores and T-scores for normative groups by age and gender are available using a standardization sample of 200 teens matched to the US Census on ethnicity and parent education within sex and age bands	Measures personal attributes critical for overcoming life obstacles. Subscales are sense of mastery (optimism, self-efficacy, adaptability); sense of relatedness (trust, support, comfort, tolerance); and emotional reactivity (sensitivity, recovery, impairment)
Behavior		
Sexual behavior (Diiorio et al., 2002, 2006)		Items include ever having had sexual intercourse, age at initiation, # of partners, and condom use (for those reporting initiation)

### Statistical Analysis

Preliminary analyses included inspection of frequency distributions and dispersion of data as well as calculations of summary statistics (mean, standard deviations, and percentages). ANOVA (continuous measures) and chi-square tests (categorical variables) were used to test whether baseline characteristics/measures were similar between Prepared and TEEN groups. The analyses followed an intent-to-treat principle; all data were analyzed by initial assignment to the treatment group, regardless of intervention exposure. Rubin’s multiple imputation method with 11 repeated imputations was employed to impute the missing endpoint for conducting the intent-to-treat analysis. Generalized linear models (GLMs) with the identity link function were used to compare Prepared and TEEN. The analysis models included an intercept term, an indicator for Prepared (vs. TEEN), time indicators for the 6-month and 12-month follow-up visits (vs. baseline), and an intervention-by-time interaction term. Generalized estimating equation (GEE) methods with identity working correlation matrix were selected to account for within-subject correlation due to multiple assessments for the same subject as well as the effect of clustering caused by the conduct of the interventions in groups (Paik, 1997). GEE, a population average model, was selected over a subject-specific model, such as random effects models, because we were interested in the impact of Prepared on overall risk reduction of HIV acquisition at the population level. If, on average, Prepared was more effective than TEEN over time, then it will be beneficial from the public health point of view to implement it in the target population. We are less interested in knowing the weighted average intervention effect while holding all the random effect variables fixed, which is the question that the subject-specific model aims to answer. Although the estimated intervention effect from two methods may be similar, their interpretations are very different. In addition, with non-normal distributions in the dependent variables, GEE is often superior to random effects models. Therefore, GEE is a preferable method for analyzing the data from both practical and statistical standpoints. The regression coefficient corresponding to the intervention-by-time interaction term estimates the difference in population mean change in outcome (from baseline to follow-up) between Prepared and TEEN, which represents the Prepared intervention effect. Intervention effects are reported with their *p* values and corresponding 95% confidence intervals. The Holm step-down procedure was employed to adjust for multiple comparisons *within* each domain (such as knowledge, beliefs) at each follow-up assessment. We also examined effectiveness by gender to explore whether the Prepared intervention is beneficial overall or just for a specific subgroup. We conducted such analyses to help interpret the findings from the analysis of the whole sample.

## Results

Of the 200 randomized to Prepared, 69.5% participated; 86.3% of these completed it (Fig. 2). Of the 197 randomized to TEEN, 78.7% participated and 74.2% completed it. In Prepared, 85.5% completed the T2 survey and 81% completed T3; in TEEN, 79.7% completed T2 and 74.6% completed T3. Rates of program participation and completion, as well as sample retention to T3, did not differ by gender or age.

### Baseline Characteristics

The mean age of youth was 13.4 years; half (50%) were female (Table 2). The sample was mostly Black (44%) and Latino (50%), and over 80% lived in poverty (received Medicaid or public assistance excluding those who could not answer). 44% lived with both biological parents and 40% lived in single-parent households. HIV knowledge was low (mean = 5.7/18). Intention to have sex in the next 6 months also was low (14%), and most (85%) reported they would definitely/probably refuse sex without a condom. Few had engaged in oral, anal, or vaginal sex (12%), and fewer (5%) had engaged in unprotected sex in the prior 6 months.

**Table 2:** Participant characteristics overall and by group assignment

	TEEN (*n* = 197) 49.6%	Prepared (*n* = 200) 50.4%	Total (*n* = 397)	*p* value
Age, mean (SD)	13.4 (.8)	13.5 (.8)	13.4 (.8)	0.26
% female	99 (50.3%)	98 (49.0%)	197 (49.6%)	0.80
Race/ethnicity				0.72
Black	84 (42.6%)	92 (46.0%)	176 (44.3%)	
Latino	101 (51.3%)	96 (48.0%)	197 (49.6%)	
White/Asian	2 (1.0%)	4 (2.0%)	6 (1.5%)	
Mix/other	10 (5.1%)	8 (4.0%)	18 (4.5%)	
Household member receives public assistance				0.36
Yes	98 (49.7%)	108 (54.0%)	206 (51.9%)	
No	18 (9.1%)	23 (11.5%)	41 (10.3%)	
Do not know	81 (41.1%)	69 (34.5%)	150 (37.8%)	
Participants’ family structure (cohabitants)				0.11
Both biological parents	77 (39.1%)	97 (48.5%)	174 (43.8%)	
Single biological parent	86 (43.7%)	73 (36.5%)	159 (40.1%)	
Biological parent plus parent’s partner	31 (15.7%)	23 (11.5%)	54 (13.6%)	
All others	3 (1.5%)	7 (3.5%)	10 (2.5%)	

### Baseline Differences Between Groups

Randomization was successful with only one significant difference between groups: TEEN started out higher on outcome expectancy for condom use.

### Change Over Time

In the longitudinal analyses comparing changes by group (GEE), those in Prepared demonstrated significantly greater improvements over those in TEEN at T2, 6 months post-intervention, in HIV knowledge (*p* < .001), sexual self-efficacy (*p* < .05), and outcome expectancy for condom use (*p* < .05). At T3, 12 months post-intervention, we found significant differences in improvement over time favoring Prepared in outcome expectancy for condom use (*p* < .001), sexual self-efficacy (*p* < .001), and intention to talk to one’s partner about HIV (*p* < .05). There were no significant differences in abstinence self-efficacy or outcome expectancies, recognizing risky gender norms, relationship expectations, or in intention to have sex, to refuse sex without a condom or to carry a condom by group at either T2 or T3 (Table 3).

**Table 3:** Model predicted mean and proportion for each group at each time point as well as the group comparisons in change over time (i.e., *d*_2_ and *d*_3_) for the Full Sample

	Baseline		T2: 6 months		T3: 12 months		*d*_2_*	*p* value *d*_2_	*d*_3_*	*p* value *d*_3_
	TEEN (*n* = 197)	Prepared (*n* = 200)	TEEN (*n* = 157)	Prepared (*n* = 171)	TEEN (*n* = 147)	Prepared (*n* = 162)				
Relationship factors										
Romantic Beliefs Scale	9.5	9.5	9.5	9.9	9.8	10.0	0.23	.40	0.16	.57
Monitoring/control	12.7	12.7	12.9	13.1	12.7	12.8	0.14	.48	0.12	.68
Openness	10.1	10.3	10.0	10.4	10.2	10.3	0.21	.31	0.04	.85
Gender norms										
Endorse male sexual risk behaviors	11.8	11.2	12.3	12.2	12.7	12.4	0.35	.47	0.24	.81
Endorse female sexual risk behaviors	9.7	9.5	8.8	9.1	9.1	8.6	0.27	.37	− 0.10	.86
Resilience (T-scores)										
Mastery (high = better mastery)	48.1	48.0	48.8	48.4	47.8	48.6	− 0.24	.78	0.92	.27
Interpersonal relationships (high = better relationships)	47.0	46.8	46.9	48.3	46.7	46.9	1.50	.24	0.33	.82
Emotional reactivity (high = worse reactivity)	50.9	49.9	50.9	49.2	51.5	51.1	− 0.68	.53	0.71	.65
Cognitive factors: behavioral intentions										
Plan to have vaginal, anal, or oral sex (“sex”)+	30 (15%)	27 (14%)	34 (22%)	29 (17%)	34 (23%)	34 (21%)	0.85	.60	1.02	.96
Plan to talk to a partner about HIV/STIs +	63 (32%)	58 (29%)	49 (31%)	59 (35%)	**40 (27%)**	**60 (37%)**	1.34	.24	**1.81**	**.02**
Would refuse sex without a condom	169 (86%)	168 (84%)	136 (87%)	144 (84%)	126 (86%)	140 (86%)	0.95	.87	1.22	.60
Plan to carry a condom	57 (29%)	66 (33%)	62 (40%)	79 (46%)	61 (42%)	75 (46%)	1.09	.70	1.01	.98
Cognitive factors: social cognitions										
HIV knowledge	5.6	5.8	**7.2**	**8.3**	7.6	8.9	**0.99**	**.00**	1.10	.14
Abstinence outcome exp.	58.6	58.8	58.1	58.9	58.4	58.8	0.51	.55	0.12	.92
Abstinence self-efficacy	62.3	63.2	67.1	66.9	67.0	68.7	− 1.13	.28	0.77	.47
Sexual self-efficacy	61.0	60.7	**64.9**	**66.8**	**63.8**	**67.3**	**2.27**	**.03**	**3.27**	**.00**
Condom outcome exp.	**35.9**	**34.6**	**35.2**	**35.4**	**34.6**	**35.1**	**1.38**	**.02**	**1.67**	**.00**

+, includes those not planning to have sex

*d_2_ and *d*_3_ represent the Prepared intervention effect for T2 and T3 respectively. For continuous outcomes, they are mean difference (between Prepared and TEEN) in change over time whereas for the dichotomous variables, they represent the ratio (between Prepared and TEEN) of two odds ratios (i.e., the change over time for Prepared and for TEEN)

Text **in bold** indicates statistical significance at *p* < .05

Both boys and girls benefitted from Project Prepared. Girls showed greater positive changes in abstinence outcome expectancies at T2 (*p* < .05) and  reduced endorsement of risky female sexual behavior at T3 (*p* < .05) . Sexual self-efficacy improved for girls in Prepared; however, TEEN girls’ scores also improved, and the groups did not differ significantly. Boys in Prepared improved in abstinence self-efficacy (*p* < .001) and condom outcome expectancy at T3 (*p* < .001); and sexual self-efficacy (*p* < .001) and intention to talk to one’s partner about HIV at T2 and T3 (*p* < .001) (see Table 4). Condom outcome expectancy declined over time for TEEN boys, especially at T3, but boys in Prepared maintained T2 program effects. There was no parallel decline in outcome expectancies for girls in either TEEN or Prepared. TEEN girls improved more in resilience (mastery) at T2 (*p*<.05) and abstinence self-efficacy at T3 (*p*<.05).

**Table 4:** Model predicted mean and proportion for each group at each time point as well as the group comparisons in change over time (i.e., *d*_2_ and *d*_3_) by each of the two gender subgroups

	**Females only**									
	**T1: Baseline**		**T2: 6 months**		**T3: 12 months**		***d***_2_*	***p*** **value** ***d***_**2**_	***d***_3_*	***p*** **value** ***d***_**3**_
	**TEEN (*N*** **= 99)**	**Prepared (*N*** **= 98)**	**TEEN (*N*** **= 80)**	**Prepared (*N*** **= 80)**	**TEEN (*N*** **= 78)**	**Prepared (*N*** **= 79)**				
Relationship expectations										
Romantic Beliefs Scale	9.3	9.5	9.8	10.0	9.9	10.5	− 0.05	.90	0.44	.28
Monitoring/control	12.9	13.0	13.2	13.1	13.0	13.0	− 0.14	.64	− 0.07	.84
Openness	10.5	10.5	10.5	10.8	10.3	10.7	0.15	.27	0.19	.50
Gender norms										
Endorse male sexual risk behaviors	11.0	10.9	11.9	12.1	13.5	12.5	0.12	.90	− 0.66	.64
Endorse female sexual risk behavior	7.8	8.2	7.9	7.7	**9.5**	**8.1**	− 0.51	.21	**− 1.37**	**.02**
Resilience (T-scores)										
Mastery	48.2	48.3	**48.6**	**47.2**	48.3	47.9	**− 1.50**	**.03**	− 0.51	.46
Interpersonal relationships	47.7	47.3	46.8	47.1	46.7	46.5	0.60	.59	0.13	.94
Emotional reactivity	51.7	50.0	50.2	51.5	52.2	52.3	3.05	.12	1.83	.52
Cognitive factors: behavioral intentions, *N* (%)										
Plan to have sex+	12 (12%)	9 (9%)	14 (18%)	10 (13%)	11(14%)	13 (17%)	0.92	.86	1.64	.31
Plan to talk to partner about HIV/STIs+	27 (27%)	25 (26%)	26 (33%)	19 (24%)	22 (28%)	19 (24%)	0.71	.35	0.88	.74
Would refuse sex without a condom	97 (98%)	92 (94%)	77 (96%)	76 (95%)	74 (95%)	73 (92%)	2.34	.43	2.08	.48
Plans to carry a condom	21 (21%)	21 (21%)	29 (36%)	26 (33%)	29 (37%)	23 (29%)	0.84	.61	0.69	.33
Cognitive factors: social cognitions										
HIV knowledge	6.0	6.2	8.3	9.3	8.7	9.5	0.80	.07	0.53	.51
Abstinence outcome exp.	65.7	63.7	**63.5**	**63.0**	64.5	61.9	**1.58**	**.02**	− 0.53	.69
Abstinence self-efficacy	66.9	68.9	72.8	73.6	**74.0**	**73.7**	− 1.30	.31	**− 2.35**	.**04**
Sexual self-efficacy	61.5	61.4	66.7	67.9	67.6	68.1	0.87	.65	0.26	.88
Condom outcome exp.	36.5	34.6	36.2	35.9	36.1	35.3	1.27	.10	0.90	.26
	**Males only**									
	**T1: Baseline**		**T2: 6 months**		**T3: 12 months**		***d***_2_*	***p*** **value d**_**2**_	***d***_3_*	***p*** **value d**_**3**_
	**TEEN (*N*** **= 98)**	**Prepared (*N*** **= 102)**	**TEEN (*N*** **= 77)**	**Prepared (*N*** **= 91)**	**TEEN (*N*** **= 69)**	**Prepared (*N*** **= 83)**				
Relationship expectations										
Romantic Beliefs Scale	9.7	9.6	9.2	9.8	9.7	9.6	0.52	.12	0.52	.12
Monitoring/control	12.5	12.4	12.7	13.1	12.4	12.5	0.40	.10	0.32	.39
Openness	9.8	10.0	9.5	10.1	10.0	10.0	0.27	.48	− 0.11	.65
Gender norms										
Endorse male sexual risk behaviors	12.5	11.4	12.7	12.2	12.1	12.3	0.59	.29	1.17	.23
Endorse female sexual risk behaviors	11.5	10.6	**9.8**	**10.3**	8.6	9.1	**1.05**	**.03**	1.23	.14
Resilience (T-scores)										
Mastery	48.0	47.7	49.0	49.6	47.2	49.4	0.85	.47	2.56	.16
Interpersonal relationships	46.3	46.3	47.0	49.3	46.7	47.3	2.21	.20	0.48	.81
Emotional reactivity	50.2	49.8	51.7	47.1	50.7	50.0	− 4.16	.11	− 0.26	.90
Cognitive factors: behavioral intentions, *N* (%)										
Plan to have sex+	18 (18%)	18 (18%)	20 (26%)	19 (21%)	23 (33%)	21 (25%)	0.79	.56	0.71	.43
Plan to talk to partner about HIV or STIs+	36 (37%)	33 (32%)	**23 (30%)**	**40 (44%)**	**18 (26%)**	**41 (49%)**	**2.24**	**.02**	**3.36**	**.00**
Would refuse sex without a condom	72 (74%)	76 (75%)	59 (77%)	68 (75%)	52 (75%)	67 (81%)	0.86	.70	1.30	.57
Plan to carry a condom	36 (37%)	45 (44%)	33 (43%)	53 (58%)	32(46%)	52 (63%)	1.37	.28	1.43	.28
Cognitive factors: social cognitions										
HIV knowledge	5.2	5.4	6.0	7.5	6.4	8.4	1.33	.07	1.82	.10
Abstinence outcome exp.	51.3	54.1	52.5	55.2	51.6	55.8	− 0.10	.92	1.45	.37
Abstinence self-efficacy	57.6	57.7	61.2	61.0	**59.0**	**63.9**	− 0.20	.90	**4.78**	**.00**
Sexual self-efficacy	60.6	60.1	**62.9**	**65.8**	**59.5**	**66.5**	**3.69**	**.02**	**6.33**	**.00**
Condom outcome exp.	35.2	34.7	34.2	35.0	**32.9**	**35.0**	1.51	.07	**2.45**	**.00**

+, includes those not planning to have sex

*d_2_ and *d*_3_ represent the Prepared intervention effect for T2 and T3 respectively. For continuous outcomes, they are mean difference (between Prepared and TEEN) in change over time whereas for the dichotomous variables, they represent the ratio (between Prepared and TEEN) of two odds ratios (i.e., the change over time for Prepared and for TEEN)

Text **in bold** indicates statistical significance at *p* < .05

### Sexual Behavior

Sexual risk behavior was not an outcome because we judged it unlikely that many participants would initiate/engage in intercourse during the project. As expected, only 53 teens reported having intercourse between months 6 and 12, too few to have the power to assess whether observed differences by group were statistically significant. However, these findings were suggestive: those randomized to Prepared reported fewer episodes of unprotected sex on average than those in TEEN (2.7 vs. 5.4).

## Limitations

This study has several limitations. First, we are skeptical about the ability of 12–14-year-olds with no sexual experience to answer self-efficacy or intention questions. For example, if youth say they have no intention of discussing condom use with a partner, they may mean that they have no intention to have sex. Second, our 1-year follow-up window was too short to observe effects of Prepared on sexual risk behavior. Third, this single-site study in one city may not be generalizable to other populations. Fourth, the study recruited participants who had primary care visits and may under-represent youth who do not have access to or use health care.

## Discussion

Project Prepared aimed to address cognitions, gender norms, resilience, and relationship factors that had been shown in the literature to influence early sexual initiation. It relied on social cognitive theories of Reasoned Action and Planned Behavior to identify the specific cognitions that were covered in the curriculum; it addressed gender norms and relationship beliefs that were associated in the literature with sexual risk behavior and identified resilience attributes that could be enhanced to provide ongoing protection against risk. Given the acknowledged sexual naiveté of the population, the curriculum covered basic information—how HIV and STIs are transmitted, how to use a condom and barriers to use, how risky sexual behavior is rooted in gender norms, and how to identify healthy and unhealthy relationships. The intensive intervention included an internship designed to put teens in the role of expert to their peers, which we believed would reinforce ownership of the content, and enhance commitment to safer sex behaviors.

Prepared demonstrated statistically significant improvements compared to TEEN in social cognitions and gender norms, which is evidence that Prepared was successful in manipulating the factors it was designed to affect. In addition, we noted improvement in behavioral intentions to engage in safer sex. Program effects were shown for both boys and girls, with effects a bit stronger for boys. Previous HIV/STI prevention programs that examined program effects by gender have reported mixed results, with some benefitting boys and others helping girls. Our data show that both girls and boys randomized to Prepared improved post-intervention, but girls in TEEN improved on some outcomes as well (with the notable exception of perspectives on risky female gender norms). This research further demonstrates that an HIV/STI intervention for early adolescents could be effectively implemented for boys and girls together. The Prepared curriculum was designed to provide lessons appropriate to both boys and girls and included lessons on gender norms and messages targeted to both male and female perspectives.

## Suggestions for Future Research

Research is needed on why boys benefited more strongly from Prepared. Girls in Prepared also improved on key variables, but girls in TEEN were nearly as likely as girls in Prepared to improve as well. It is unlikely that this is due to unintended effects of TEEN, which provided no sexual or reproductive health information. It is possible that girls in this age range are more likely than boys to receive formal or informal instruction on HIV, STIs, and relationships in school, from their families, or elsewhere. Although statistically the benefits of Prepared accrued more to boys, girls’ scores improved as much as boys’ over time, therefore, we believe that it is warranted to recommend the use of Prepared for both genders. There is a notable lack of published successful interventions with adolescent boys, indicating the promise of Prepared to fill an important gap; however, further research is necessary to identify successful mediators within interventions with this population (Picot et al., 2012).

A large literature reports attenuation of the effects of HIV/STI prevention programs. Prepared demonstrated significant effects on cognitive factors over 12 months—social cognitions and behavioral intentions. However, given the developmental changes in adolescence; the variety in experiences between younger and older boys and girls in dating, sex, and condom use; and the challenges adolescents have in making healthy choices concerning their sexual and reproductive risk, we believe that interventions for young teens may not be sufficient to guide youths’ sexual decisions as they enter middle adolescence. Sexual risk reduction interventions are offered on an “immunization” model; i.e., once the skills and knowledge are delivered in a program, it is assumed that the teen is protected long term. However, this assumption should be tested; research is needed on the utility of long-term, sustained intervention that evolves developmentally with teens, and targets information to the sexual evolution of adolescents’ experience (Dinaj-Koci et al., 2015). These interventions would begin early, emphasizing basic information and anticipatory guidance and over time offer increasingly sophisticated sexual and reproductive health information; condom and communication skills; relationship skills; and access to teen-friendly medical care, including STI and HIV testing and pre-exposure prophylaxis. Research on the benefits of a K through 12 approach to sexual education might be the solution to the problem that effects of risk reduction programs attenuate after the programs end.

## Supplementary Information

ESM 1 (DOC 219 kb)
